# Interleukin 8 is a biomarker of telomerase inhibition in cancer cells

**DOI:** 10.1186/s12885-018-4633-x

**Published:** 2018-07-09

**Authors:** Peter Solomon, Yuying Dong, Shaillay Dogra, Romi Gupta

**Affiliations:** 1 Department of Pathology, Yale University School of Medicine, LH-306, New Haven, CT 06510 USA; 2 Singapore Institute of Clinical Sciences, Agency for Science Technology and Research (A*STAR), Brenner Centre for Molecular Medicine, 30 Medical Dr., Singapore, 117609 Singapore

**Keywords:** Telomerase, Interleukin, Viability, Biomarker, Response

## Abstract

**Background:**

Telomerase activity is required for both initiation and maintenance of tumorigenesis and over 90% cancers overexpress telomerase. Therefore, telomerase targeting has emerged as a potential strategy for cancer treatment. In agreement with this, several telomerase inhibitors are being tested for cancer treatment and have shown some promise. However, because of the variability in response between the cancer patients, it is important to identify biomarkers that allow for distinguishing cancers that are responsive to telomerase inhibition from the cancers that are not. Therefore, in this study we performed experiments to identify a biomarker that can be used to predict telomerase inhibition induced tumor growth inhibition.

**Methods:**

In our study, we have performed transcriptome-wide gene expression analysis on multiple ovarian and colon cancer cell lines that were treated with telomerase inhibitor imetelstat and were responsive to telomerase inhibition-induced tumor growth attenuation.

**Results:**

We demonstrate that telomerase inhibition by telomerase inhibitor imetelstat results in decreased expression of interleukin 8 (IL8) in all telomerase responsive cancer cell lines. This phenomenon is of general occurrence because we find that multiple ovarian and colon cell lines show decrease in IL8 mRNA and protein levels after telomerase inhibition. Additionally, we find loss of IL8 phenocopy Telomerase inhibition mediated growth inhibitory effect in cancer cells.

**Conclusion:**

Taken together, our results show that IL8 is a biomarker that predict telomerase inhibition mediated growth attenuation of cancer cells and its loss phenocopy telomerase inhibition. Therefore, IL8 expression can be utilized as a biomarker for telomerase targeted cancer therapies to potentially predict therapeutic response.

**Electronic supplementary material:**

The online version of this article (10.1186/s12885-018-4633-x) contains supplementary material, which is available to authorized users.

## Background

Telomeres are region of repetitive nucleotide sequences at the end of a chromosome that protects chromosomal ends from fusion or from being recognized as damaged DNA [1–3]. In normal cells, during every DNA replication cycle, the telomere ends become shorter due to the end replication problem, which consequentially results in replicative senescence [4–6]. Telomerase is an RNA-protein enzyme complex responsible for maintaining telomere length and telomerase activity is required for both initiation and maintenance of human cancers [7–9]. Telomerase is activated in approximately 90% of tumors and is an important event in cellular immortalization process [8, 10, 11]. The mechanisms of telomerase overexpression in cancer cells are still not clear. Some studies propose that telomerase promoter can acquire mutations that result in generation of new transcription factor binding sites and activation of telomerase [12]. Furthermore, since over 90% of cancer types express telomerase, telomerase represents an important target for cancer therapy [13–15]. However, telomerase targeting-based cancer therapies have thus far provided only limited success [16–19], which highlights the importance of identifying the biomarker that might predict the response to telomerase inhibition.

In our previous study, we have shown that simultaneous inhibition of CDKN1A and telomerase by imetelstat (JNJ-63935937, also known as GRN163L) leads to synergistic tumor growth inhibition [20]. In this study, we aimed to identify the biomarker that can predict response to telomerase inhibition-based therapy. To do so, we performed transcriptome-wide gene expression analysis on multiple ovarian and colon cancer cell lines that were treated with telomerase inhibitor imetelstat and were responsive to telomerase inhibition-induced tumor growth attenuation. Imetelstat is a potent and specific telomerase inhibitor and in clinical trials for cancer treatment [21, 22]. We find that upon treatment with telomerase inhibitor imetelstat, expression of IL8 mRNA and protein is inhibited in multiple ovarian and colon cancer cells. We also show that IL8 inhibition also suppresses the cancer cell viability. These results indicate that inhibition of IL8 upon telomerase inhibition by imetelstat act as biomarker to predict the response to telomerase-based therapy.

## Methods

### Cell culture

HCT116 cells were a kind gift of Bert Vogelstein (Johns Hopkins Medical School). COLO205 (ATCC® CCL-222™) and OVCAR5 cells (obtained as part of NCI-60 cell line panel, NCI) were obtained from American Type Culture Collection (ATCC) and were grown as recommended. Cells were treated with 2.5 μM imetelstat or mismatch oligonucleotide (Geron Corporation) twice per week for up to 6 weeks and did not exceed 80% confluence during the treatment. IL8 (GFP tagged) cDNA ORF was obtained from origene. Reagents RPMI 1640 (Catalog no. # 11879020, Thermofischer scientific), DMEM (Catalog no. # 11966092, Thermofischer scientific), Penicillin-streptomycin (Catalog no. # 15140122, Thermofischer scientific) and Fetal Bovine serum (Catalog no. # 10082147, Thermofischer scientific) were purchased from Thermofischer scientific.

### Transfections, shRNAs and preparation of lentiviral particles

*IL8* and control non-specific shRNAs were obtained from Open Biosystems. Table 1 shows the product IDs for all shRNAs. Lentiviral particles were prepared by co-transfecting the shRNA plasmids and lentiviral packaging plasmids, pSPAX2 and pMD2.G, into 293 T cells using Effectene (Qiagen) and following the protocol at the Broad Institute’s website (http://www.broadinstitute.org/rnai/public/resources/protocols).

### Cell viability, Colony formation assay and tumorigenesis assays

To measure cell viability two kinds of assays were performed. For Trypan blue exclusion viability assays, cells were plated and were treated with mismatch oligo’s or imetelstat for 2 weeks triplicate. After 2 weeks of treatment they were trypsinized and were with mixed with an equal volume of Trypan Blue Solution (Invitrogen) and counted using Countess (Invitrogen). Relative cell viability of imetelstat treated cells with respect to control mismatch oligo treated cells is plotted. For MTT assay cells are plated in in 96 well plate. Then they were either treated with mismatch oligo’s or imetelstat for 2 weeks. After treatment, 10μl of 5 mg/ml of MTT is added to the medium each well and incubated for 1 h. Medium is removed and 100μl of DMSO is added, mixed well and the measurement is performed at absorbance 590 and 630 nm wavelength. Average is taken and the reading at 690 is subtracted form that at 590. Relative cell viability of imetelstat treated cells with respect to control mismatch oligo treated cells is plotted. For colony formation assays, 10^3^ cells were seeded in triplicate. Then they were either treated with mismatch oligo’s or imetelstat for 2 weeks. Colonies formed were stained with 0.005% crystal-violet solution and counted. For in vivo experiment, Eight-week old, athymic nude (NCr nu/nu) mice (*n* = 5) were injected subcutaneously with cancer cells (2.5 × 10^6^). After one week, tumor-bearing mice received mismatch oligonucleotide or imetelstat (30 mg/kg bodyweight) three times per week by intraperitoneal injection. Tumor growth was measured every week using calipers, and tumor volumes were calculated using the formula 0.5 X length X width^2^ for every week.

### Quantitative RT-PCR and immunoblot analysis

qRT-PCR and Immunoblot was performed as described in [20]. Briefly For mRNA expression analyses, total RNA was extracted with TRIzol (Invitrogen) and purified using RNAeasy mini columns (Qiagen), and cDNA was generated using M-MuLV first-strand cDNA synthesis kit (New England Biolabs) as per manufacturer’s instructions. Quantitative RT-PCR was performed using Power SYBR-green kit (Applied Biosystems) for mRNA expression analysis or the miScript SYBR-green PCR assay kit (Qiagen), as per manufacturer’s instructions. Actin was used as an internal control. For Immunoblotting whole cell protein extracts were prepared using IP lysis buffer (Pierce) containing Protease Inhibitor Cocktail and Phosphatase Inhibitor Cocktail (Sigma-Aldrich, St.Louis, MO). Protein concentration was estimated using a Bradford Assay kit (Bio-Rad). Proteins separated on 10% or 12% polyacrylamide gels were transferred to PVDF membranes using a wet transfer apparatus from Biorad. Membranes were blocked with 5% skim milk and probed with primary antibodies followed by the appropriate secondary HRP-conjugated antibody (GE healthcare, UK). Blots were developed using the Supersignal Pico Reagent. Antibody and primer information is provided in Table 1.

### TRAP assay and telomere length measurement

The TRAP assay was performed essentially as described [23]. Every plate included standards, inactivated samples and lysis buffer as controls. Each sample was analyzed at least in triplicate. Telomerase activity was plotted relative to control mismatch oligonucleotide treated cells. Telomere length measurement was performed using Relative Human Telomere length Quantification qPCR assay kit from Science cell (Catalog no. # 8908). The assay was performed as described by the supplier.

### Transcriptome-wide gene expression measurement assay and data analysis

For microarray experiments using HCT116, OVCAR5 and COLO205 (5 million) cells were treated with imetelstat for 2 weeks, total RNA was isolated from the cells as described above and used to generate labelled antisense RNA. All antisense RNAs were made using the Ambion MessageAmp Kit and hybridized to Illumina HumanHT-12 V4.0 expression BeadChip using Illumina’s protocol.

The microarray data were processed using GenomeStudio™ (Illumina), log2-transformed, and quantile-normalized using the “lumi” package of Bioconductor. All samples passed quality-control (QC) assessment, which included checking various control plots as suggested by Illumina, as well as other standard microarray-related analyses. Differential expression analyses were performed using the “limma” package, and a moderated t-test with a Benjamini-Hochberg multiple testing correction procedure was used to determine statistical significance (adjusted *P*-value, < 0.05). Pathway analysis of differentially expressed genes for each comparison was performed using MetaCore™ (version 6.8 build 29,806, GeneGo). Microarray data were submitted to Gene Expression Omnibus. Geo accession number is GSE106539.

### Statistical analysis

All the experiments were conducted in three biological replicates. The results for individual experiments were expressed as mean ± SEM. The *p*-values were calculated using t-test by using GraphPad Prism version 6.0 h for Macintosh, GraphPad Software, San Diego California USA (www.graphpad.com).

## Results

### Identification of telomerase inhibition responsive Cancer cell lines

To identify biomarker of telomerase-inhibitor therapy, we analyzed a series of cancer cell lines of different tissue origin (Table 2). To this end, we treated these cancer cell lines, with imetelstat, a telomerase inhibitor. Imetelstat is a synthetic lipid-conjugated, 13-mer oligonucleotide N3’ P5’-thio-phosphoramidate and is complementary to the template region of telomerase RNA (hTR). Inside the cells, imetelstat acts as a competitive enzyme inhibitor that binds and blocks the active site of the enzyme (a “telomerase template antagonist”) thus inducing growth inhibition [24, 25].

**Table 1:** Primer sequences for RT-qPCR analysis; clone ID and catalog numbers for shRNAs (Open Biosystems); antibodies used

Application	Gene symbol	Forward primer (5′-3′)	Reverse primer (5′-3′)
RT-qPCR	IL8	taaaaagccaccggagcact	atcaggaaggctgccaagag
	actin	gccgggacctgactgactac	tcttctccagggaggagctg
	Gene symbol	Clone ID	Catalog number
shRNAs	IL8	TRCN0000058028	RHS3979–9625212
		TRCN0000058030	RHS3979–9625214
Antibodies	Protein	Source	Dilution
	IL8	Santa Cruz Biotechnology	1:500 dilution
	GFP	Santa Cruz Biotechnology	1:500 dilution
	Actin	Cell Signaling	1:1000 dilution
Inhibitor	Concentration	Source	
*Imetelstat*	2.5 μM	Geron Corporation	
*Mismatch Oligonucleotide*	2.5 μM	Geron Corporation

**Table 2:** Cell lines and their tissue origin

S. No.	Cell lines	Tissue Origin
1	SW480	Colon
2	KM12	Colon
3	HCT116	Colon
4	COLO205	Colon
5	OVCAR3	Ovary
6	ADR-RES	Ovary
7	SKOV3	Ovary
8	OVCAR5	Ovary

**Fig. 1:**
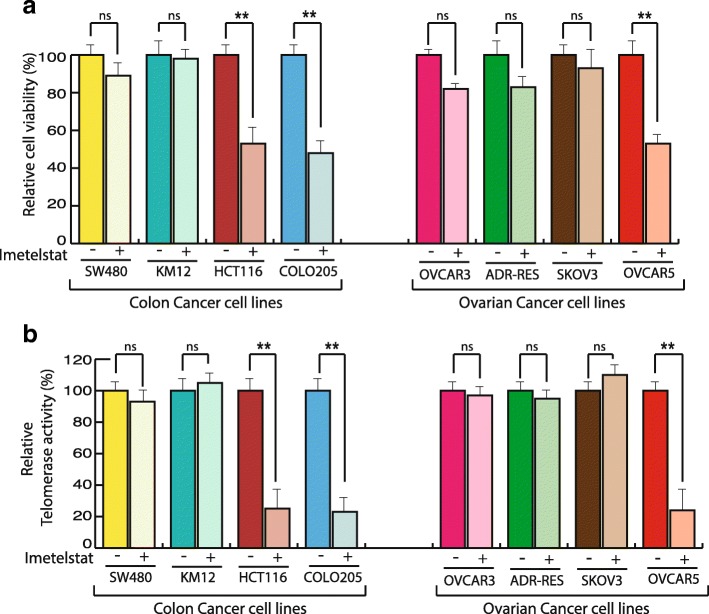
Measurement of response of cancer cells to telomerase inhibition. Indicated cell lines were treated with mismatch oligonucleotide nucleotide or imetelstat for 2 weeks. **a** Cell viability was monitored by trypan blue exclusion assay and relative cell viability with respect to mismatch oligonucleotide nucleotide treated cell is plotted (**b**) Telomerase activities was measured by TRAP assay and relative telomerase activity with respect to mismatch oligonucleotide nucleotide treated cell is plotted. Error bar shows Standard Error Mean (SEM). (**, *p* < 0.001), (ns, non-significant)

We first optimized the imetelstat concentration for the treatment of various cancer cell lines. To do so, we treated different cancer cell lines with multiple concentrations of imetelstat for determining IC50. We found that IC50 for imetelstat for different cancer cell lines was in the range of 2.5μM as shown in Additional file 1: Figure S1. Next, we treated multiple ovarian and colon cancer cell lines of different tissue origin for 2 weeks with 2.5μM imetelstat and identified HCT116, COLO205 and OVCAR5 as three cancer cell lines that showed strong growth inhibition following imetelstat treatment and also showed concomitant decrease in telomerase activity (Fig. 1**).** We additionally checked telomere length in the cell lines that showed growth inhibition upon imetelstat treatment and found that HCT116, COLO205 and OVCAR5 show shortened telomere length upon imetelstat treatment as compared to control treated cells (see Additional file 2: Figure S2). These results demonstrate that inhibition of telomerase in ovarian and colon cancer cell lines leads to their growth attenuation.

### Transcriptome-wide gene expression analysis identifies IL8 as a biomarker that predict response to telomerase inhibition

**Fig. 2:**
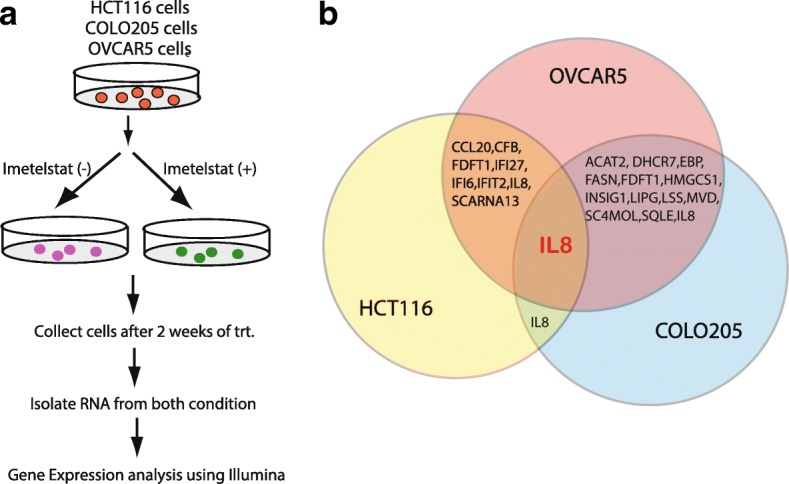
Gene expression analysis using Illumina identified IL8 as a common biomarker of telomerase response. **a** Schematics of the Transcriptome-wide gene expression analysis is described. It was performed in HCT116, OVCAR5 and COLO205 cells. Briefly equal number of cells from these cell lines were either untreated or treated with imetelstat for 2 weeks after which RNA was isolated and candidate genes that were either upregulated or downregulated were identified. **b** Venn diagram showing the common genes that were identified after imetelstat treatment in between two cell lines and also between three cell lines

In order to identify the genes that can function as biomarker to predict response to telomerase-based therapy, HCT116, COLO205 and OVCAR5 cell lines that showed strong growth inhibition following imetelstat treatment were treated with imetelstat for 2 weeks and then transcriptome-wide gene expression analysis was performed (Fig. 2a**)**. We observed multiple genes that were upregulated and downregulated in each cancer cell line (Fig. 2b**)**. We also performed analysis to identify the common genes that were altered upon imetelstat treatment in all these cancer cell lines. Our results showed that in all three cancer cell lines, IL8 was the only common candidate that was downregulated in imetelstat treated cells as compared to control cells (Fig. 2b**)**. These results in sum indicate that the cancer cells that show response to telomerase inhibition downregulate IL8 levels and this phenomenon is of general occurrence as multiple ovarian and colon cancer cell lines that undergo growth inhibition upon imetelstat shows IL8 downregulation (Fig. 2b**)**.

### Telomerase inhibition suppresses cancer cell viability

**Fig. 3:**
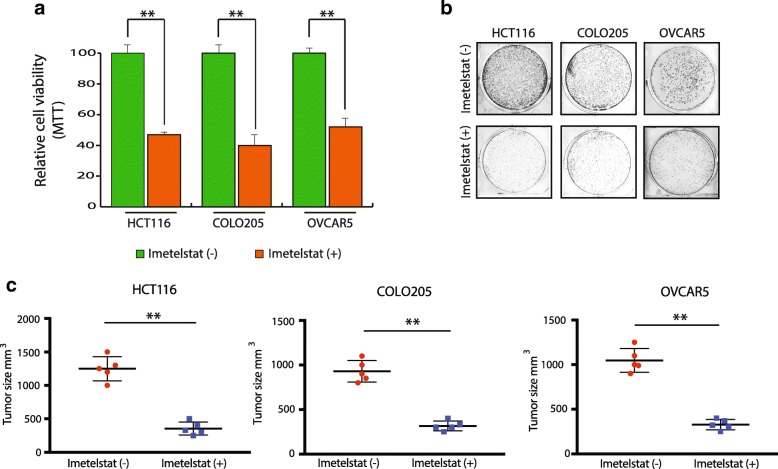
Telomerase inhibition suppresses cancer cell viability. **a** Cell viability was measured by MTT assay. Relative cell viability is plotted in 2 weeks imetelstat treated cells in comparison to control mismatch oligonucleotide treated cells for the shown cell lines. **b** Indicated cell lines were treated with mismatch oligonucleotide or imetelstat for 6 weeks and stained with crystal violet. Representative wells are shown. **c** HCT116, OVCAR5 and COLO205 cells were injected subcutaneously in athymic nude mice. These were either treated with either mismatch oligonucleotide or imetelstat for 4 weeks. Average tumor volumes for indicated cell lines after 4 weeks of treatment are shown. Error bar shows Standard Error Mean (SEM). ** represents *p* < 0.001

In Fig. 1, we have shown the effect of imetelstat on cancer cell viability using trypan blue exclusion assay. In order to confirm the growth inhibitory effect of imetelstat on HCT116, COLO205 and OVCAR5 cells we employed an additional assay called MTT assay. MTT is a calorimetric based assay to detect metabolic activity of cell which is dependent of the number of viable cells. To do so, we treated HCT116, COLO205 and OVCAR5 cells with imetelstat for 2 weeks and then performed MTT assay as described in method section. As expected, we found that after two weeks of imetelstat treatment HCT116, COLO205 and OVCAR5 cells showed significant growth inhibition (Fig. 3a**)**. This was further confirmed by colony formation assay as shown in Fig. 3b. Next, in order to study the effect of imetelstat on the growth of these cancer cell lines in vivo, we performed xenograft-based mouse tumorigenesis assay. Our results showed that imetelstat significantly inhibited the growth of HCT116, COLO205 and OVCAR5 tumors upon imetelstat treatment (Fig. 3c). These results confirmed that the inhibition of telomerase affects the cancer cell growth. Therefore, expression of telomerase is critical for cancer cell survival and serves as an effective target to inhibit cancer cell growth.

### Telomerase inhibition decreases both IL8 mRNA and protein level

**Fig. 4:**
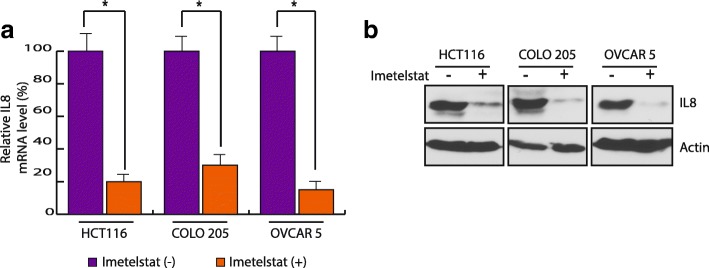
IL8 levels decrease upon inhibition of telomerase. Indicated cells were treated with imetelstat for 2 weeks. **a** IL8 RNA level was measured by qRT-PCR. Actin was used as internal control. Relative levels with respect to control mismatch oligonucleotide treated cell is plotted. **b** IL8 protein level was measured by immunoblotting. Actin was used as loading control. Error bar shows Standard Error Mean (SEM). (*, *p* < 0.01)

After confirming the cancer cells growth suppression upon telomerase inhibition, we measured IL8 transcript and protein level in HCT116, COLO205 and OVCAR5 cells after imetelstat treatment and found that both IL8 mRNA as well as IL8 protein level decreases upon imetelstat treatment (Fig. 4). This was observed in multiple ovarian and colon cancer cell lines suggesting that decrease in IL8 level is a common biomarker predictive of telomerase inhibition induced cancer cells growth suppression (Fig. 4). We also checked IL8 levels in the cell lines that did not show growth suppression upon telomerase inhibition and found that in these cell lines IL8 level did not change upon imetelstat treatment, as shown in Additional file 3: Figure S3. These results in sum indicate that decrease in IL8 level is a specific biomarker for the cancer cell lines that shown growth suppression upon telomerase inhibition.

### IL8 inhibition affects the cell viability and its overexpression rescues telomerase inhibition induced growth inhibitory effect

**Fig. 5:**
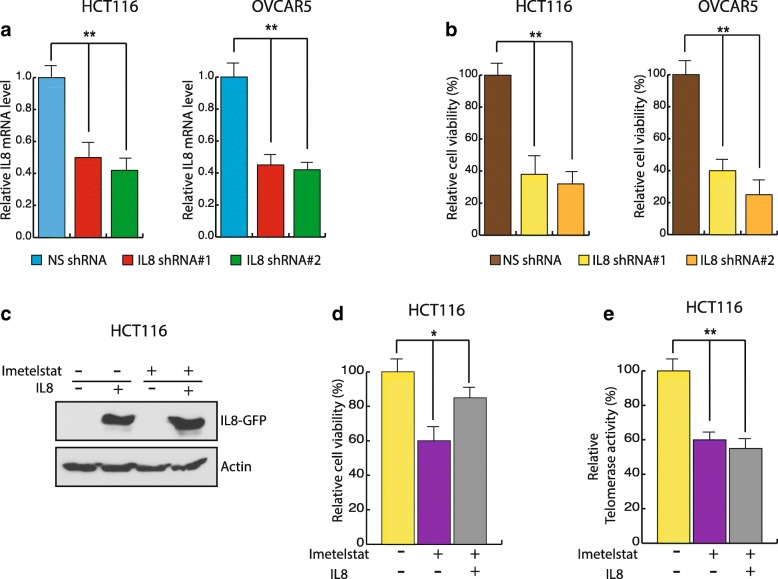
IL8 inhibition phenocopy telomerase inhibition. **a** HCT116 and OVCAR5 cell lines stably expressing a non-specific (NS) shRNA or *IL8* shRNAs. Knockdown is determined by measuring IL8 mRNA levels and plotting with respect to the control cell expressing nonspecific shRNA. **b** Cell viability of the cells expressing either nonspecific or IL8 shRNA was measured by trypan blue exclusion assay. Cell viability relative to control cell expressing nonspecific shRNA is plotted. HCT116 cells were either treated with mismatch oligonucleotide or imetelstat for 2 weeks and were then transfected to overexpress IL8-GFP tagged cDNA. **c** Western blot for GFP tag was performed to check IL8 overexpression in the cells. **d** Cell viability was measured by trypan blue exclusion assay and plotted with respect to control mismatch oligonucleotide treated cells. **e** Telomerase activities was measured by TRAP assay and plotted with respect to control mismatch oligonucleotide treated cells. Error bar shows Standard Error Mean (SEM). (**, *p* < 0.001 and *, *p* < 0.01)

Our result showed that IL8 level decreases upon imetelstat treatment. To confirm if the decrease in IL8 levels leads to decrease in cell viability, we knocked down IL8 expression using shRNAs in different cancer cell lines (Fig. 5a). Next, these cells were checked for cell viability. As shown in Fig. 5b*,* we find that the cells expressing IL8 shRNA have lower cell viability as compare to control cells expressing non-specific shRNA. In order to conclusively show the inhibition of IL8 is involved in telomerase inhibition induced growth inhibitory effect, we overexpressed IL8 in imetelstat treated cells (Fig. 5c), and then checked the cell viability. We found that IL8 overexpression rescued telomerase inhibition induced growth inhibitory effect (Fig. 5d). This was not due to restoration of telomerase activity upon IL8 expression, because no change in telomerase activity was observed after IL8 over expression in imetelstat treated cells (Fig. 5e). Taken together, these results led us to conclude that telomerase inhibition leads to decreases IL8 levels, which can be employed as a biomarker for predicting response to telomerase-based therapy in cancer.

## Discussion

Early diagnosis and identification of new predictive and diagnostic biomarker has helped to determine the effectiveness of various therapies and the treatment response and predicting outcome of cancer treatment more accurately [26–28]. Overexpression of telomerase enzyme and consequential immortalization is a key step for cancer initiation and progression. Furthermore, telomerase has been shown to be necessary for maintaining tumor growth. Therefore, many inhibitors that suppress telomerase expression are currently under investigation for cancer treatment [29–31]. However, because of variability between the patient response to telomerase inhibition, identification of biomarkers that might predict cancers cell response to telomerase inhibitor will provide immense clinical benefits.

**Fig. 6:**
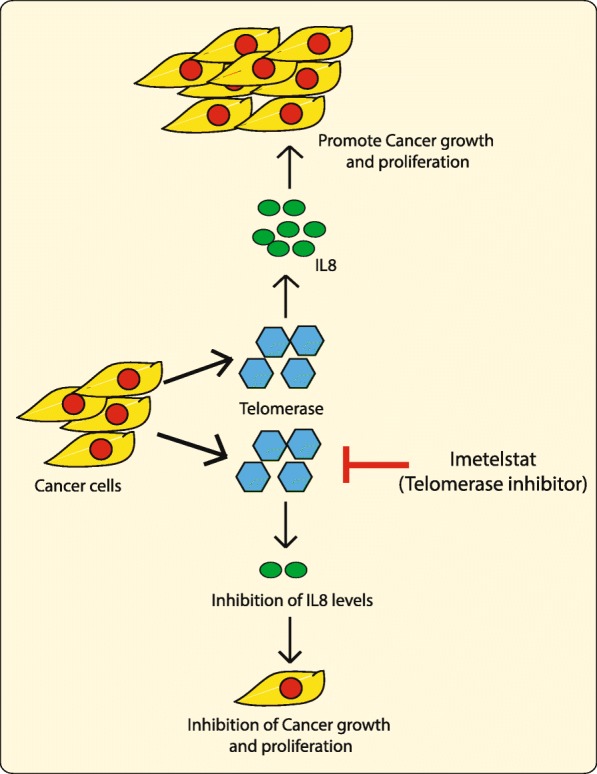
IL8 is a biomarker that could predict telomerase inhibition response. Cancer cells exclusively produce enzyme telomerase which is now targeted with pharmacological inhibitors like imetelstat. Inhibition of Telomerase leads to inhibition of IL8, which is a pro-oncogenic cytokine and thus inhibits cancer cells growth and progression. IL8 act as predictive biomarker for telomerase response

In our previous study, we showed that simultaneous inhibition of CDKN1A and telomerase by imetelstat leads to synergistic tumor growth inhibition [20]. In the current study, we have made an attempt to identify the biomarker that could predict telomerase inhibition response and to do that we performed gene expression microarray analysis on multiple ovarian and colon cancer cell lines that were responsive to telomerase inhibitor imetelstat treatment. Our results are summarized in Fig. 6 and discussed below.

The results shown in our study is more practical and advantageous because it’s not based on hypothesis-based biomarker discovery. Our study is largely discovery-based biomarker identification, where we have employed unbiased high through-put based Transcriptome-wide gene expression analysis to discover a functional predictive biomarker of telomerase inhibition response. We have further employed secondary assays to validate and confirm our findings in multiple ovarian and colon cancer cell lines.

In our study, we show that different cell lines respond differently to telomerase inhibition. Next, we find that the cell lines that show growth inhibition phenotype upon telomerase inhibition, downregulate IL8 cytokine expression level. This phenomenon is of general occurrence as we find that multiple ovarian and colon cell lines show decrease in level of both IL8 mRNA and protein upon treatment with imetelstat. Additionally, we find that this phenomenon is specific for the cancer cell lines that show strong growth inhibition following imetelstat treatment along with concomitant decrease in telomerase expression. A previous study has shown that telomerase is bound to the promoters of a subset of NF-κB target genes, including IL6, IL8, and TNF-α and stimulate their expression to sustain inflammation and promote cancer progression [32]. These studies provide us an insight into possible mechanism by which inhibition of telomerase leads to decrease in IL8 levels. Based on the previous studies that document that telomerase is directly bound to IL8 promoter, in our studies we find that non-responder cancer cell lines which don’t show significant decrease in telomerase level upon imetelstat treatment, also do not show any decrease in IL8 levels, again reconfirming that decrease in IL8 is dependent on telomerase inhibition. Therefore, this is a specific phenomenon observed only in cancer cell line that show telomerase inhibition induced growth suppression.

IL8 is a chemokine and is shown to be produced form macrophages and other type of cells [33]. Previous studies have shown that cancer cell over-express IL8 and its overexpression is associated with poor prognosis, increase in cell invasion that promotes cancer cell progression, angiogenesis, and metastases [34–36]. In fact, treatment based on inhibition of IL8 expression is shown to enhance the efficacy of the cancer-based therapies [37, 38]. Furthermore, our results show that inhibition of IL8 upon imetelstat treatment leads to inhibition of cell growth and proliferation, thus mimicking growth inhibition phenotype induced upon telomerase inhibition. In conclusion, our study identifies IL8 as an important biomarker that predict the effectiveness of telomerase-based therapy for treating cancer.

## Conclusion

Our study employs gene expression analysis to identify a new biomarker that could predict the response to telomerase inhibition. This information can be utilized in clinical settings to determine whether patient will be responsive to telomerase-based therapy or not. There are many reliable methods that allows convenient and precise measurement of IL8 levels and hence it can be effectively utilized in clinics. Furthermore, future studies are needed to identify other such predictive biomarker that will facilitate to determine the effectiveness of various therapies and treatment response and predicting outcome of cancer treatment more accurately.

## Additional files

Additional file 1: **Figure S1.** IC50 determination or Imetelstat. HCT116 and OVCAR5 cells were treated with various concentrations of Imetelstat. Cell viability was measured after 48 h of treatment by trypan blue exclusion assay. Error bar shows Standard Error Mean (SEM). ** represents *p* < 0.001. (PDF 817 kb).

Additional file 2: **Figure S2.** Relative Telomere length measurement in multiple ovarian and cancer cell lines before and after Imetelstat treatment. HCT116, COLO205 and OVCAR5 cells were either treated with control mismatch oligo or Imetelstat for 2 weeks. Relative telomere length was measured using Relative Human Telomere length Quantification qPCR assay kit from Science cell. Error bar shows Standard Error Mean (SEM). * represents *p* < 0.01. (PDF 242 kb).

Additional file 3: **Figure S3.** IL8 level determination by immunoblotting in multiple ovarian and cancer cell lines before and after Imetelstat treatment. Cancer cells were either treated with control mismatch oligo or Imetelstat for 2 weeks. IL8 protein level was measured by immunoblotting. Actin was used as loading control. (PDF 1171 kb).

## Abbreviations

CDKN1A: Cyclin dependent Kinase Inhibitor 1A
IL8: Interleukin 8
TRAP: Telomeric Repeat Amplification Protocol
