# Supplementary figures and images for: Interleukin 8 is a biomarker of telomerase inhibition in cancer cells
Source: BMC Cancer. 2018 Jul 9;18:730. doi: 10.1186/s12885-018-4633-x (PMC6038317; doi:10.1186/s12885-018-4633-x)

Figure S1

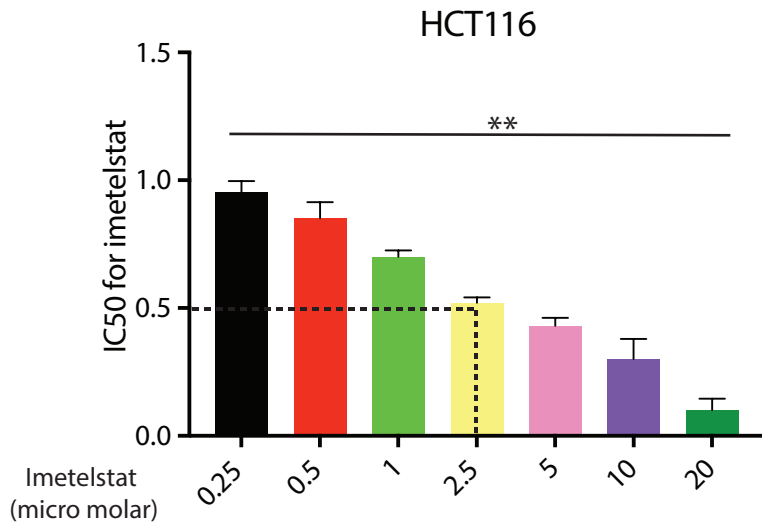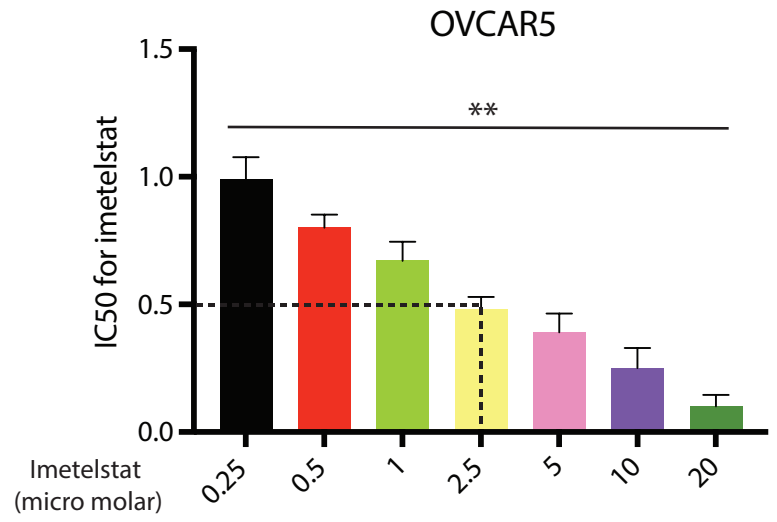

Supplement: Supplementary file 1 — Figure S1. IC50 determination or Imetelstat. HCT116 and OVCAR5 cells were treated with various concentrations of Imetelstat. Cell viability was measured after 48 h of treatment by trypan blue exclusion assay. Error bar shows Standard Error Mean (SEM). ** represents p < 0.001. (PDF 817 kb). [file 12885_2018_4633_MOESM1_ESM.pdf]

Figure S2

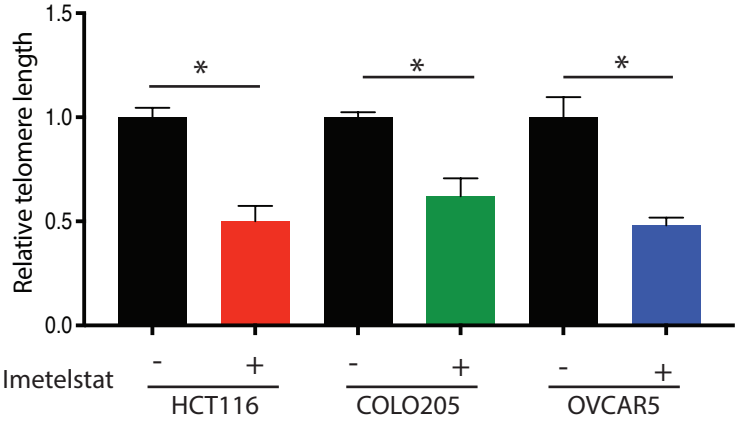

Supplement: Supplementary file 2 — Figure S2. Relative Telomere length measurement in multiple ovarian and cancer cell lines before and after Imetelstat treatment. HCT116, COLO205 and OVCAR5 cells were either treated with control mismatch oligo or Imetelstat for 2 weeks. Relative telomere length was measured using Relative Human Telomere length Quantification qPCR assay kit from Science cell. Error bar shows Standard Error Mean (SEM). * represents p < 0.01. (PDF 242 kb). [file 12885_2018_4633_MOESM2_ESM.pdf]

Figure S3

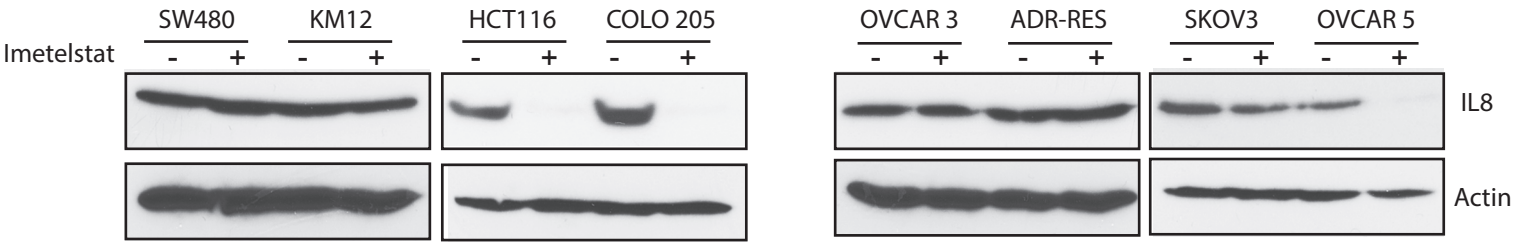

Supplement: Supplementary file 3 — Figure S3. IL8 level determination by immunoblotting in multiple ovarian and cancer cell lines before and after Imetelstat treatment. Cancer cells were either treated with control mismatch oligo or Imetelstat for 2 weeks. IL8 protein level was measured by immunoblotting. Actin was used as loading control. (PDF 1171 kb). [file 12885_2018_4633_MOESM3_ESM.pdf]
